# Management of Pacemaker Implantation during COVID-19 Infection

**DOI:** 10.1155/2020/8833660

**Published:** 2020-10-24

**Authors:** Luigi Rivetti, Roberto Mantovan, Nadir Sitta, Ricarda Marinigh, Giuseppe Allocca, Moemen Mohammed, Fausto Pizzino, Giuseppe Nucifora

**Affiliations:** Department of Cardiology, “S. Maria dei Battuti” Hospital, AULSS 2 Veneto, Via Brigata Bisagno, Conegliano (TV), Italy

## Abstract

The management of device implantation during the COVID-19 infection has not well defined yet. This is the first case of complete atrioventricular block in a symptomatic patient affected by the COVID-19 infection treated with early pacemaker implantation to minimize the risk of virus contagion.

## 1. Introduction

Heart disease patients with the concomitant COVID-19 infection could have higher risk of mortality, and their management could be particularly challenging [1].

Recently, the European Society of Cardiology (ESC) Guidance [2] and Italian position paper [3] about the treatment of cardiovascular (CV) disease in COVID 19 infection patients have been published; however, the suggested management of atrioventricular (AV) conduction disorder patients is not matching between the two proposed guidelines. This is the first report about an AV block patient affected by the COVID-19 infection treated with early permanent pacemaker (pPM) implantation.

On March 12^th^ 2020, a 75-year-old female, with no previous cardiovascular history or previous drugs taking, was admitted to our Emergency Department because of a traumatic syncope during fever. Five days prior to her admission, she had myalgia, cough, dyspnea, and fevers up to 39.5°C. At the electrocardiogram (ECG), the patient showed complete AV block with ventricular escape rhythm and atrial fibrillation. The laboratory tests revealed normal high-sensitivity troponin and ionemia, but high C-reactive protein (11 mg/l; n.v.<1 mg/L). The echocardiogram showed normal biventricular function, while the chest X-ray revealed bilateral consolidations. The moderate hypoxia (PO2 = 64 mmHg) at the blood gas confirmed the hypothesis of a pneumonia; thus, a nasopharyngeal swab was performed, with a positive result for SARS-CoV-2 on the real-time reverse transcriptase–polymerase chain reaction assay.

**Figure 1:**
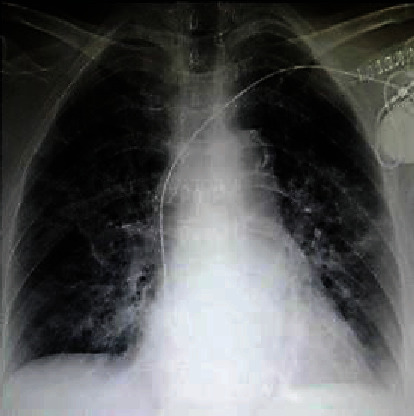
The chest X-ray of the COVID-19 patient after the pPM implantation.

**Figure 2:**
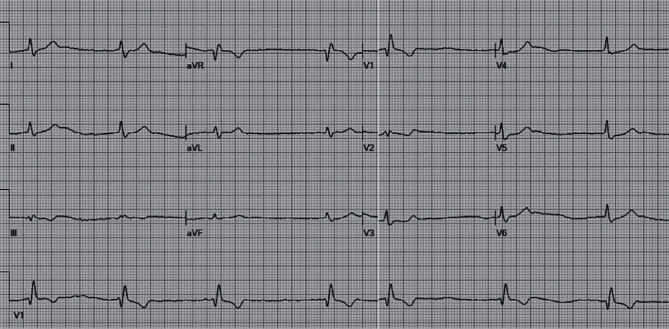
The ECG of the patient before the pPM implantation.

**Figure 3:**
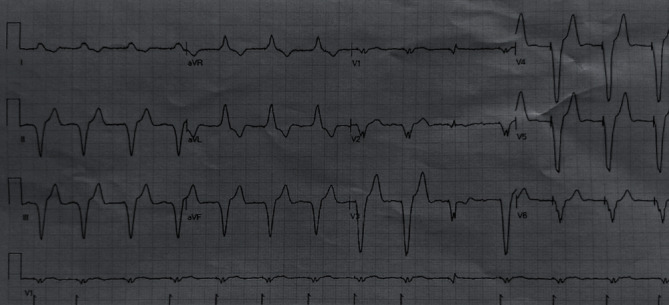
The ECG of the patient after the pPM implantation.

Although the consensus to prevent cardiac implantable electronic device (CIED) infections recommends to delay the procedure until a patient has afebrile for at least 24 hours [4], a single chamber PM (Medtronic-Advisa SR MRI™) was implanted in the same day of the admission (Figure 1), two hours after the positive result for the SARS-CoV-2 swab infection. The procedure was performed in thirty minutes using resorbable sutures to avoid further surgical wound checks. The medical staff wore FPP2-masks, while the patient had a surgical mask according to the World Health Organization recommendation [5]. At last, the patient has been transferred in a COVID 19 dedicated hospital to continue the appropriate medical therapy. There, the patient was treated with antiviral drugs (lopinavir/ritonavir 200/100 bis in die (BID) for 14 days), hydroxychloroquine (400 mg BID for the day 1 followed by 200 mg BID for four days), azythromycin (500 mg on day 1, followed by 250 mg for seven days), and enoxaparin (4000 units/day subcutaneously). After two weeks from the pPM implantation, the COVID-19 infection was effectively treated, and the patient was discharged home. No pPM-related complications were reported, as far as no other viral contagion episodes were documented among the noninfected staff. Moreover, the home-remote monitoring was preferred to check the pPM parameters after the discharge avoiding any potential favourable condition of the virus hospital reinfection. Nowadays, the patient is asymptomatic, her clinical general conditions are satisfactory, and the pPM parameters are in range. Figures 2 and 3 showed the ECG before the pPM implantation and after the COVID 19 infection. It confirmed that no change in the conduction disturbance happened when the infection was solved; moreover, the pacing rate was 100% at the PM check after 3 months.

## 2. Discussion

In the era of COVID-19, the management of heart disease patients with the concomitant virus infection has not been completely defined yet. Moreover, as other known cardiotropic virus in case of myocardial involvement [6], the hypothesis that COVID-19 could lead to the exacerbation of conduction system disorders, or sinus node disease or new-onset high degree AV block, is actually under study, albeit no cases have been already described. The hypothesis of myocarditis has been considered unlikely in this case, as far as both the high-sensitivity troponin and the biventricular function were normal, although exclusive involvement of the conduction tissue cannot be excluded. Conversely, the hypoxia due to the pneumonia has probably leaded to the demonstration of a preexisting conduction disorder in this patient.

Several guidelines have already proposed different approaches about the management of AV conduction disorders patients; however, no real-world data are available until now in this specific setting. Hence, the ESC recommendation [2] suggested a medical drug approach with isoprenaline and atropine and the implantation of temporary PM (tPM), leading the potential pPM after recovery from the COVID-19 infection. Conversely, the Italian position paper [3] recommends avoiding in any cases the tPM for risk infection, thus preferring early pPM implantation. In this controversial field, at least three reasons could explain the approach that we chose of implanting early a pPM: first of all, the early pPM implantation has reduced the time of exposure of the potential viral contagion for the medical staff, allowing us to transfer the patient to a dedicated COVID-19 hospital where continuing the appropriate drug treatment. Secondly, the early implantation of a pPM has reduced the risk of potential hemodynamic consequences for the patient, as bradycardia and cardiogenic shock, frequently due to the AV block. Thirdly, although the consensus to prevent CIED infections recommend generally to wait for 24 hours since an afebrile patient [4], it is needful to evaluate case by case, especially in viral diseases that rarely have been associated to the CIED infection yet.

Interestingly, it is known that tPM patients are up to 2.5 times more prone to develop an infection before the pPM implantation [7], with higher mean reported complication rates [8], such as lead's dislodgement with malsensing/malpacing, pneumothorax, and cardiac perforation. Moreover, it has been documented that in survivors, the duration of COVID-19 viral shedding was in media 20 days, up to a maximum of 37 days [9]. Based on these considerations, the choice of a tPM would expose not only the patient to a higher risk of complications for such a long time but also the entire staff to a higher risk of viral contagion, not allowing the immediate transfer of the patient to the dedicated COVID-19 hospital. Furthermore, in our region, the management of COVID-19 patients in dedicated areas has been also assigned to general medical doctors [10]; thus, it would have been hard for them if the specific management of a tPM is without any previous experience.

To our knowledge, this is the first report of a COVID-19 infection patient symptomatic for AV block treated with early pPM implantation. This approach allowed us to preserve all the medical equipment and the other patients from the potential viral contagion in a no COVID-19 hospital and to obtain the best outcome for the patient, transferring her to a dedicated COVID-19 area to continue the appropriate medical treatment.
